# Effects of Various Polymeric Films on the Pericarp Microstructure and Storability of Longan (cv. Shixia) Fruit Treated with Propyl Disulfide Essential Oil from the Neem (*Azadirachta indica*) Plant

**DOI:** 10.3390/polym14030536

**Published:** 2022-01-28

**Authors:** Muhammad Rafiullah Khan, Chongxing Huang, Rafi Ullah, Hakim Ullah, Ihsan Mabood Qazi, Taufiq Nawaz, Muhammad Adnan, Abdullah Khan, Hongxia Su, Liu Ren

**Affiliations:** 1 School of Light Industry and Food Engineering, Guangxi University, Nanning 530004, China; suhongxia@st.gxu.edu.cn (H.S.); liuren2020@126.com (L.R.); 2 Department of Agriculture, University of Swabi, Swabi 25130, Pakistan; rafiullah@uoswabi.edu.pk (R.U.); hakimullah277@gmail.com (H.U.); 3 Department of Food Science and Technology, The University of Agriculture Peshawar, Peshawar 25000, Pakistan; drqazi@aup.edu.pk (I.M.Q.); taufiqnawaz85@gmail.com (T.N.); 4 Guangxi Key Laboratory of Sugarcane Biology, State Key Laboratory for Conservation and Utilization of Subtropical Agro-Bioresorces, Guangxi University, Nanning 530004, China; adnan.gxu786@gmail.com (M.A.); abdullahgxu@gmail.com (A.K.)

**Keywords:** longan, fruit, polymeric films, antioxidant activity, enzymatic browning, neem, propyl disulfide, microbial decay, essential oil

## Abstract

Plant extracts represent a rich repository of metabolites with antioxidant and antimicrobial properties. Neem (*Azadirachta indica*) is a medicinal plant considered the tree of the 21st century. In this study, we investigated the antioxidant and antimicrobial effects of propyl disulfide (PD), a major volatile compound in neem seed, against the pericarp browning (BI), microbial decay incidence (DI), and water loss of longan fruit. Fresh longan cv. Shixia samples were packaged in oriented polypropylene (OPP) and polyethene (PE) packages of different thicknesses (20, 40, and 60 µm). Sterile gauze was fixed inside the packages and 500 uL of PD was placed on them to avoid the direct contact of PD with fruit samples. Packages were sealed immediately to minimize vaporization and stored at 12 ± 1 °C for 18 days. Fruit samples packaged in open net packages served as controls. The results showed that fruit treated with PD in OPP and PE packages significantly prevented losses of water, DI, and BI compared to control treatment. PD also maintained the color, TSS values, TA values, pH values, high peel firmness, high TPC content, and high TFC content, and reduced the activity levels of PPO and POD. Scanning electron microscope (SEM) analysis indicated that the exocarp, mesocarp, and endocarp of longan peel were smooth, uniform, and compact with no free space compared to control, where crakes, a damaged and loose structure, and a lot of fungal mycelia were found. The shortest shelf life of 9 days was observed in control as compared to 18 days in OPP-20 and OPP-40; 15 days in OPP-60, PE-20, and PE-40; and 12 days in PE-60 packaging films. Therefore, PD as a natural antioxidant and antimicrobial agent, in combination with OPP-20 and OPP-40 polymeric films, could successfully be applied commercially to extend the postharvest shelf life of longan.

## 1. Introduction

Longan (*Dimocarpus longan* L.) is an attractive subtropical fruit of the evergreen tree of the Sapindaceae family. The fruit is widely cultivated in many countries, especially China, Thailand, Vietnam, and Australia. The fruit has high nutritional value and is best when eaten fresh. However, longan is non-climacteric; the fruit is harvested at optimum maturity and does not continue to ripen once harvested. The fruit matures in high temperature and humidity, meaning it deteriorates rapidly once harvested due to pericarp browning and microbial rot. Further, due to the unique pericarp structure of longan fruit, the dehydration and microbial invasion expedite the senescence and browning, consequently shortening its postharvest life [1]. Several studies have been conducted on treatments such as chlorine dioxide [2]; adenosine triphosphate (ATP) [3]; hydrogen peroxide [4]; chitosan [5]; SO_2_, ClO_2_, or their combination [6]; thymol coatings and thymol fumigation [7,8]; and many other studies in order to preserve the quality and extend the shelf life. However, due to consumer awareness of the health concerns regarding the residues of the synthetic compounds and the resistance of the microbes to the existing preservatives, there is a need to develop other preservatives that are safe to humans and the environment. Therefore, the continuous use of synthetic compounds needs to be eliminated to ensure the availability of safe and fresh fruits for longer periods of time.

Recently, plant-based extracts have attracted much more interest from researchers due to their biologically active components with antioxidant and antimicrobial properties. Various essential oils have been extracted from plants and have been utilized in the food industry. The neem plant (*Azadirachta indica*) is a rich source of about 300 primary and secondary metabolites, which possess antifungal, antibacterial, and antioxidant properties [9,10,11]. In another study, more than 140 biologically active compounds have been isolated from different parts [9], which have anti-inflammatory, antihyperglycemic, antiulcer, antimalarial, antifungal, antibacterial, antioxidant, antimutagenic, and anticarcinogenic properties [10,12,13]. So far, most studies have been conducted on neem extracts in the pharmaceutical industry or traditional medicines. Few studies have reported on controlling plant diseases. Propyl disulfide compound is the major volatile compound in neem plant seeds, and our research group previously assessed the antifungal activity levels of propyl disulfide, which effectively inhibited the mycelial growth of fungi, which causes anthracnose [14] and stem end rot [15] in mango fruit, obtaining very promising results. Zakawa et al. [16] studied the effects of neem leaf extract on the fungi causing anthracnose in wild mango. In 2011, Suleiman [17] reported on the effects of neem leaf extract against tomato anthracnose.

Neem extracts have also been used to preserve a wide range of other food products. For example, Serrone et al. [11] preserved the quality of fresh retail meat using neem oil and reported its efficacy against a wide range of bacterial populations. In another study, neem cake oil was used to preserve the quality of fresh retail meat [18]. The antioxidant activity of neem oil was reported in regard to beef lipid oxidation reactions, which extended the shelf life of raw beef patties to 11 days at 4 °C [19].

Enzymatic browning, microbial decay, and water loss are the major concerns in longan fruit; therefore, in this study, we focused on the antioxidant and antimicrobial activity levels of propyl disulfide from neem. Different packaging films were tested to find the best storage conditions. A very simple, cost-effective, and practical method was proposed for PD fumigation. The in-depth antioxidant mechanism of propyl disulfide was assessed regarding the enzymatic browning reaction in longan fruit, which includes phenolic substrates, enzymes, and browning. Pearson’s correlation coefficient analysis was carried out to find the relationships between these parameters. The effect of PD on the unique pericarp structure of longan fruit and its three components were analyzed via scanning electron microscopy and are reported in detail in this paper.

## 2. Materials and Methods

### 2.1. Fumigation and Fruit Treatment

**Figure 1:**
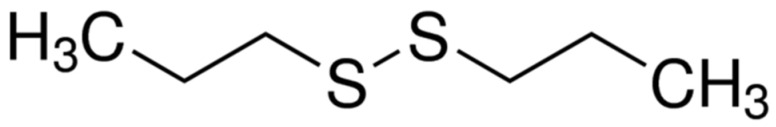
Structure of propyl disulfide.

Longan fruit cv. Shixia were harvested from a commercial farm in the Guangxi region and transported to the laboratory. Fruit samples of uniform size and color with no defects were prepared. About 500 g of fruit was packed in oriented polypropylene (Jiahang OPP Packing Bag Co., Ltd., Jinhua, Zhejiang, China) of different thicknesses (OPP-20, OPP-40, OPP-60) and polyethylene (Bailiheng PE Packaging Bag Co., Ltd., Shenzhen, China) (PE-20, PE-40, PE-60) packages. A sterile gauze was fixed inside in each package, and 500 uL of the propyl disulfide (a major volatile compound in neem (*Azadirachta indica*) seed; >99% Food grade, Aladdin Reagent Co., Ltd., Shanghai, China; Figure 1) was placed on the gauze to avoid its direct contact with the fruit. Each package was immediately sealed to reduce the evaporation of the compound from the bag. For comparison, fruit samples packaged in open net packages without propyl disulfide were considered the control treatment. All packages were stored at 12 ± 1 °C. The quality of the longan fruit was evaluated every third day using three bags (three replicates) from each treatment.

### 2.2. Quality Evaluation Tests

#### 2.2.1. Weight Loss, Decay Incidence, and Pericarp Browning Index

Weight loss was determined by weighing the fruit on day 0 and then every third day, and the results were expressed as percentages. Fruit showing visible decay symptoms on the surface were considered decayed, and all the decayed fruit were counted and the decay percent was calculated using the following formula:

(1) $$\mathrm{Percentage}\;\mathrm{of}\;\mathrm{fruit}\;\mathrm{decay}\;=\frac{\mathrm{Number}\;\mathrm{of}\;\mathrm{decayed}\;\mathrm{fruit}}{\mathrm{Total}\;\mathrm{number}\;\mathrm{of}\;\mathrm{fruit}\;}\times 100$$

The pericarp browning index (BI) of longan fruit was determined by observing the amount of total brown area on each fruit surface, whereby 1 = 0% (no browning), 2 = 1–10% (slight browning), 3 => 10–25% (moderate browning), 4 => 25–50% (severe browning), 5 => 50%, calculated using the below equation [20]. Fruit with a browning scale above 3 was the limit of acceptability:

(2) $$\mathrm{BI}=\sum^{\;}\frac{\mathrm{Browning}\;\mathrm{level}\;\times \mathrm{number}\;\mathrm{of}\;\mathrm{fruit}\;\mathrm{at}\;\mathrm{each}\;\mathrm{browning}\;\mathrm{level}}{\mathrm{Total}\;\mathrm{number}\;\mathrm{of}\;\mathrm{fruit}\;\mathrm{in}\;\mathrm{the}\;\mathrm{treatment}}$$

#### 2.2.2. Color, Firmness, Total Soluble Solids, Titratable Acidity, and pH

Pericarp color (L*, a*, b*) was measured using a Konica Minolta Spectrophotometer (CM-3600d, Konica Minolta Sensing Inc., Tokyo, Japan) using 3 fruit samples from each replicate. The firmness of the peel was measured using a texture analyzer (TA.XT Plus 10752, Godalming, UK) in compression mode. A 2-mm-diameter plunger was used to puncture the fruit to a depth of five mm at a speed of 20 mm min^−1^. The maximum force needed to penetrate the fruit was recorded in newtons (N). A total of 3 fruit samples were used for each test from each replicate. For TSS, TA, and pH, the flesh of about 15 fruit samples from each replicate was ground, filtered through muslin cloth, and clear juice was obtained. TSS was determined using a digital Abbe refractometer (Way-2S, Shanghai Shenguang Instruments and Instrument Co. Ltd., Shanghai, China. Titratable acidity (TA) was carried out via the titration of juice with 0.1 N NaOH. The pH of the longan juice was determined using a pH meter (FE28, Mettler Toledo Co. Ltd., Shanghai, China). TSS, TA, and pH were conducted in triplicate.

#### 2.2.3. Extraction and Determination of Phenolic and Flavonoid Contents

Phenolic and flavonoid contents were extracted from the pericarps of 15 fruit samples according to Khan et al. [20]. Reaction mixtures for TPC and TFC were prepared according to the methods of Khan et al. [20] and Dewanto et al. [21], respectively, using a UV–visible spectrophotometer (SPECORD 50 Plus, Analytik Jena, Germany). The results for TPC and TFC were expressed as milligrams of gallic acid per kg and milligrams of catechin per kg of fresh weight, respectively. The experiment was performed in triplicate.

#### 2.2.4. Polyphenol Oxidase (PPO) and Peroxidase (POD) Activity Levels

The antioxidant effect of PD on the inhibition of enzymes activity levels was determined using the pericarps of 15 fruit samples. Crude enzyme extract was obtained according to the method used by Khan et al. [7] and Duan et al. [22]. The reaction mixture used to assess the PPO activity was prepared by following the protocol used by Khan et al. [7] and Jiang [23]. Absorbance was recorded at 410 nm for 5 min with a UV–visible spectrophotometer (SPECORD 50 Plus, Analytik Jena, Germany). One unit of enzyme activity was defined as the activity that caused a change of 0.001 in the absorbance per min. The reaction mixture for POD activity was prepared following the protocol used by Khan et al. [7] and Zhang et al. [24] and absorbance was measured using a UV–visible spectrophotometer (SPECORD 50 Plus, Analytik Jena, Germany). One unit of enzyme activity was defined as the amount that caused a change of 0.01 in the absorbance per min. Protein contents were determined according to the method used in [25]. Enzyme (PPO and POD) activity levels were expressed as units min^−1^ mg^−1^ protein. The experiments were performed in triplicate.

### 2.3. Scanning Electron Microscope and Pericarp Microstructure

Longan pericarps were prepared by following the protocols of Yao et al. [1] and Chitbanchong et al. [26], with slight modifications. Briefly, pericarps of 3 mm squares were washed twice with 0.1 M of phosphate buffer (pH 7.4). The pieces were immediately shifted to the 2.5% glutaraldehyde prepared in 0.1 M of phosphate buffer and kept overnight for 24 h at 4 °C. Samples were then rinsed in the same buffer and postfixed in 1% osmium tetroxide for 2 h and stepwise exposed to a series of ethanol–buffer mixtures of 30%, 50%, 70%, 80%, 90%, and 100% ethanol for 15 min for dehydration. The dried samples were then mounted on specimen stubs, sputter-coated with gold, and viewed under SEM (Inspect^TM^, FEI company, Hillsboro, OR, USA) with an accelerating voltage of 20 kV.

### 2.4. Statistical Analysis

Data were subjected to analysis of variance (ANOVA) using Statistix. The least significant difference (LSD) tests were performed to determine the significant differences (*p* ≤ 0.05) among the treatments. The correlation analysis was carried out in Microsoft Excel 2016.

## 3. Results

### 3.1. Weight Loss, Decay Incidence, and Pericarp Browning

**Figure 2:**
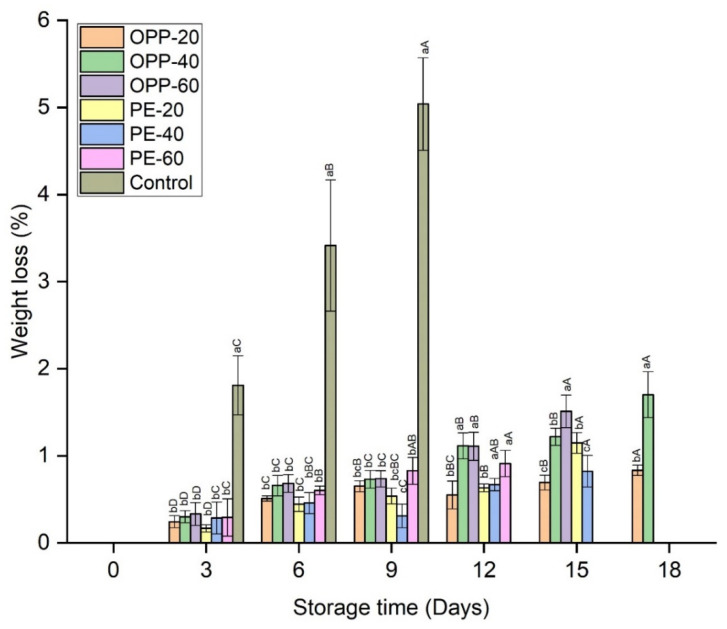
Weight loss rates (%) of longan fruit samples in different packaging films fumigated with propyl disulfide and control, stored at 12 ± 1 °C. Vertical bars represent means ± standard deviations (*n* = 3). Different small and capital letters show significant differences between the treatments within the same day and between the days in the same treatment, respectively, via LSD test at *p* ≤ 0.05.

**Figure 3:**
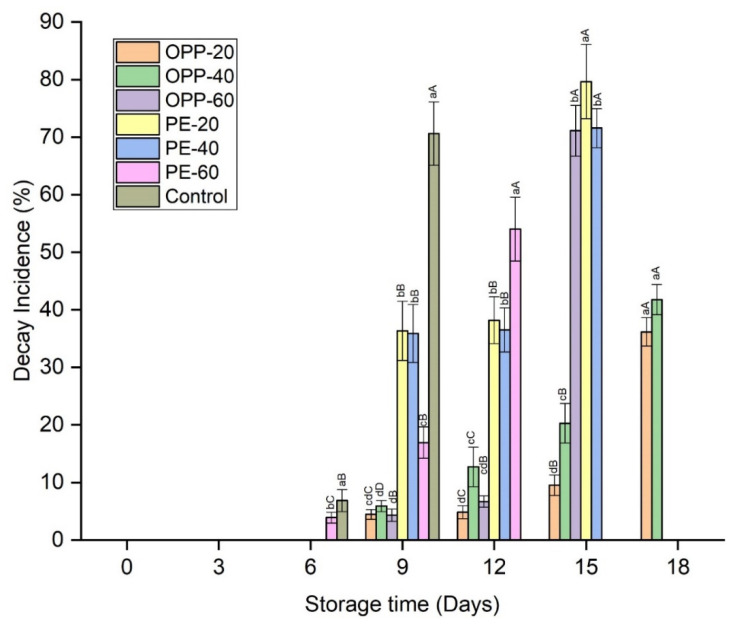
Decay incidence rates (%) of longan fruit samples in different packaging films fumigated with propyl disulfide and control, stored at 12 ± 1 °C. Vertical bars represent means ± standard deviations (*n* = 3). Different small and capital letters show significant differences between the treatments within the same day and between the days in the same treatment, respectively, via LSD test at *p* ≤ 0.05.

**Figure 4:**
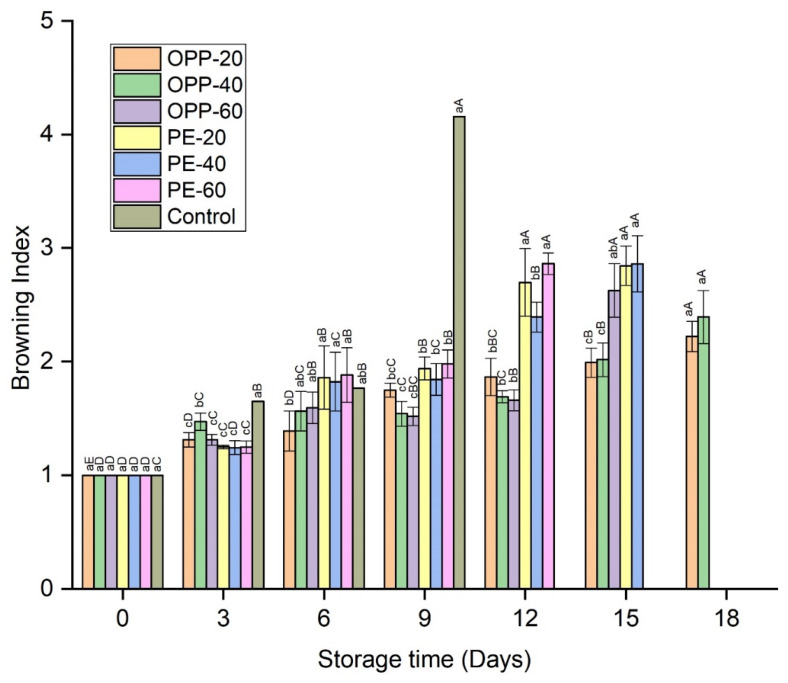
Pericarp browning rates of longan fruit samples in different packaging films fumigated with propyl disulfide and control, stored at 12 ± 1 °C. Vertical bars represent means ± standard deviations (*n* = 3). Different small and capital letters show significant differences (*p* ≤ 0.05) between the treatments within the same day and between the days in the same treatment, respectively, via LSD test at *p* ≤ 0.05.

An increase in weight loss was observed with storage time. Weight loss was significantly higher in control samples throughout the storage time, while in all other packaged samples no significant differences were observed until day 9 (Figure 2). From day 12 onward, some differences in weight loss were observed. Among all the treatments, the lowest weight loss was found in OPP-20. Statistics within the storage intervals also showed that weight loss increased significantly on each quality test day in each treatment; however, OPP-20 comparatively prevented the loss in weight more than other treatments (Figure 2). No decay symptoms were found in any treatment until day 6, except PE-60 and control, which showed decay rates of 3.91% and 6.87%, respectively. On day 9, DI increased in all treatments, being significantly (*p* ≤ 0.05) higher in the control, followed by PE-20 and PE-40. Due to the high DI rate, the control treatment was discarded on day 9 (70.62%), while the storage of PE-60 and OPP-60 and PE-20 and PE-40 treatments was discontinued on day 12 and day 15, respectively (Figure 3). Among all treatments, the lowest DI rates were observed in OPP-20 and OPP-40, which extended the longan storage life to up to 18 days (Figure 3). Pericarp browning (BI) was gradually enhanced in all treatments. BI was significantly (*p* ≤ 0.05) higher in control followed by PE treatments. Among the OPP films, OPP-20 and OPP-40 maintained lower BI rates than all other packaging films during the entire storage period (Figure 4).

### 3.2. Firmness of Longan Fruit Samples

**Figure 5:**
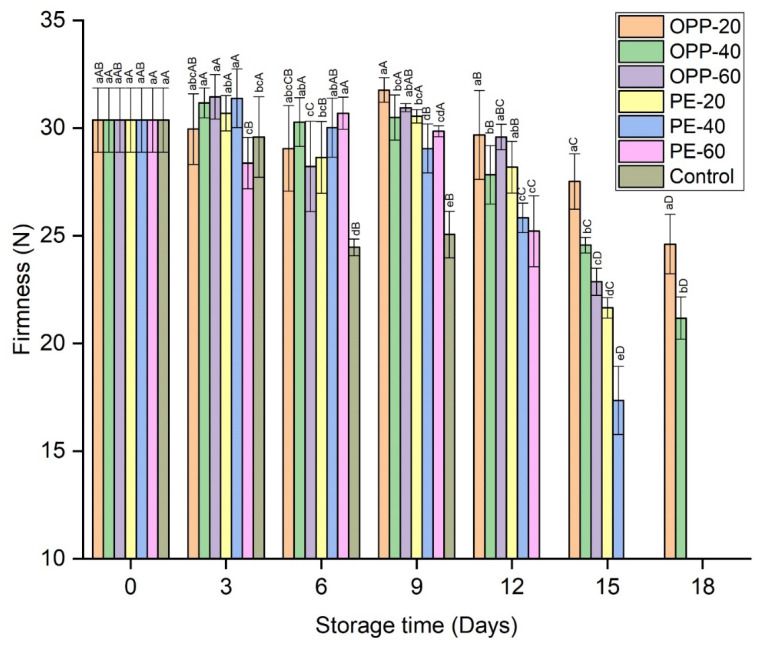
Peel firmness rates of longan fruit samples in different packaging films fumigated with propyl disulfide and control, stored at 12 ± 1 °C. Vertical bars represent means ± standard deviations (*n* = 6). Different small and capital letters show significant differences between the treatments within the same day and between the days in the same treatment, respectively, via LSD test at *p* ≤ 0.05.

The peel firmness of longan fruit samples decreased with the extension of storage time. The peel firmness significantly declined in the control treatment. Among the other treatments, the firmness fluctuated and dropped at the end of storage time in each treatment, probably associated with decay incidence. In comparison to other films, OPP-20 maintained the highest level of firmness, followed by OPP-40 (Figure 5).

### 3.3. Color (L*, a*, and b*) Values

**Figure 6:**
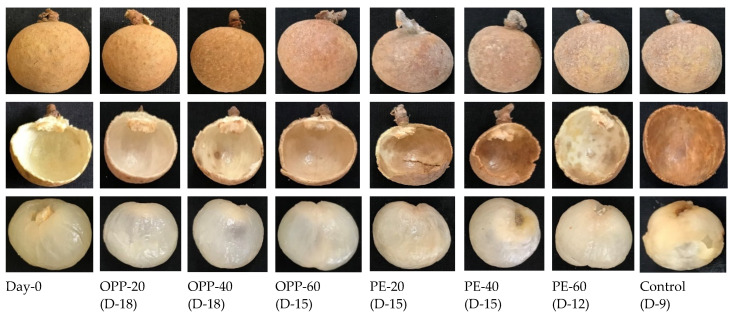
Changes in the peel and flesh of longan fruit samples during storage at 12 ± 1 °C treated with propyl disulfide and packaged in oriented polypropylene (OPP-20, OPP-40, and OPP-60), polyethylene (PE-20, PE-40, and PE-60), and control (open net) packages.

The lightness (L*) values of the longan pericarps were reduced in all treatments with storage time, as shown in Table 1. The decrease in L* values in the control treatment was comparatively high compared to other packaging films, and storage was discontinued on day 9. Similarly, the a* values, which indicate the redness of fruit samples, increased over time, and high a* values were obtained in the control treatment. The b* values, which indicate the yellowness of the longan pericarp, decreased with storage. Statistical analysis within treatments and storage intervals revealed the greatest decrease in b* values in the control treatment (Table 1). With prolonged storage time, the changes in pericarp color became more prominent, as presented in Figure 6. The outer and inner pericarps of fruit samples changed to dark brown with storage, with more severe effects in control fruit (day 9) than other treatments.

### 3.4. Total Phenolic (TPC) and Total Flavonoid Contents (TFC)

**Figure 7:**
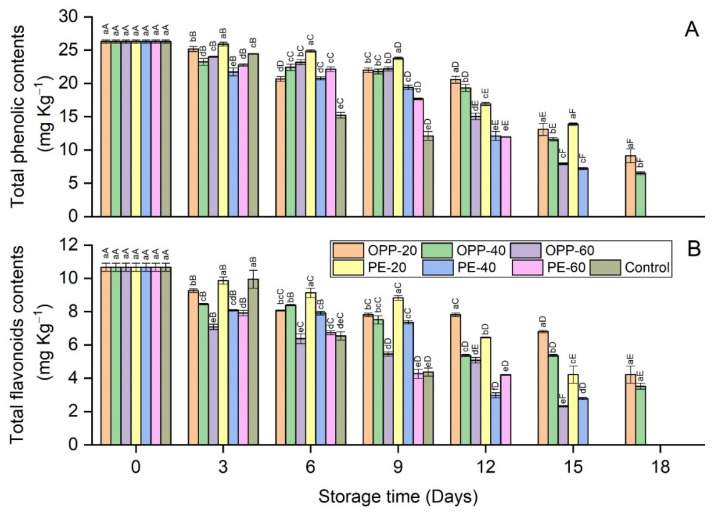
Total phenolic (**A**) and total flavonoid (**B**) contents in longan fruit samples in different packaging films fumigated with propyl disulfide and control, stored at 12 ± 1 °C. Vertical bars represent means ± standard deviation (*n* = 3). Different small and capital letters show significant differences between the treatments within the same day and between the days in the same treatment, respectively, via LSD test at *p* ≤ 0.05.

Figure 7A,B presents the total phenol and flavonoid contents in the longan fruit samples. A continuous decline was observed in all treatments in terms of TPC and TFC contents, being significantly (*p* ≤ 0.05) higher in control samples. The contents of phenols and flavonoids varied slightly among the treatments during storage; however, OPP-20 and PE-20 showed relatively higher TPC and TFC contents than the other treatments.

### 3.5. Enzymes Activity Levels

**Figure 8:**
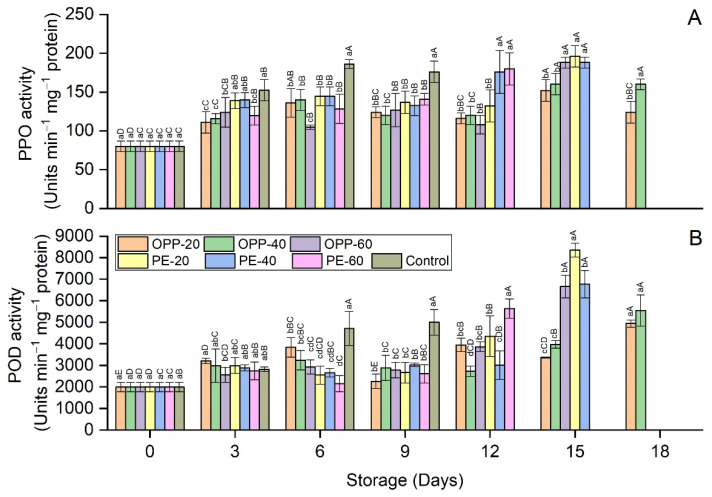
Polyphenol oxidase (**A**) and peroxidase (**B**) activity levels in longan fruit samples in different packaging films fumigated with propyl disulfide and control, stored at 12 ± 1 °C. Vertical bars represent means ± standard deviations (*n* = 3). Different small and capital letters show significant differences between the treatments within the same day and between the days in the same treatment, respectively, via LSD test at *p* ≤ 0.05.

Enzyme activity levels are presented in Figure 8A,B. Polyphenol oxidase (PPO) and peroxidase (POD) activity levels significantly increased in all treatments with storage time. The highest activity levels for PPO and POD were found in control treatment at days 6 and 9. Generally, at the end of the storage period, PPO and POD activity levels were higher in OPP packages than PE packages. The longest shelf life times were obtained for OPP-20 and OPP-40 packages with no difference in PPO and POD activity levels, with few exceptions (Figure 8A,B).

### 3.6. TSS, TA, and pH

In all treatments, TSS decreased with increased storage time (Table 2). No significant differences were found between the treated and control fruit samples, with few exceptions. Similarly, packaging films did not affect the TA and pH values, as no significant differences were found in the treated and control fruit samples, with few exceptions. However, storage time had an effect, as TA and pH values increased in all treatments with increasing storage time (Table 2).

### 3.7. Scanning Electron Microscopy and Pericarp Microstructure

**Figure 9:**
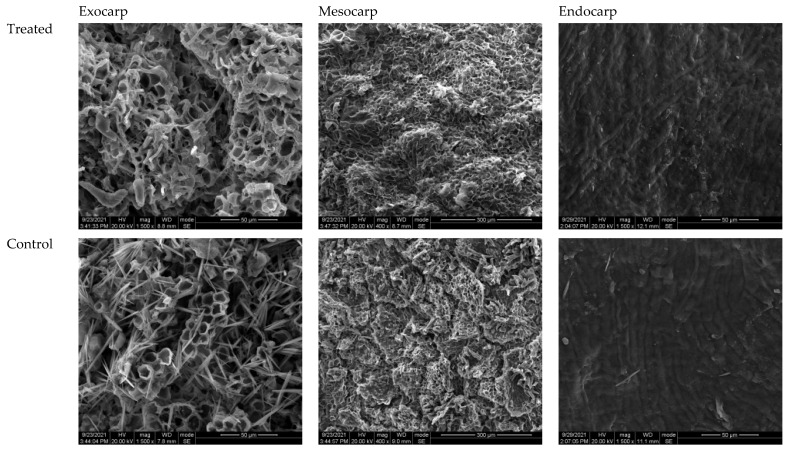
Scanning electron micrographs of treated and control longan pericarp. Exocarp samples at 1500× magnification, mesocarp samples at 400× magnification, and endocarp samples at 1500× magnification.

Longan peel consists of three major parts, namely the exocarp, mesocarp, and endocarp. In this experiment, SEM analysis of the longan fruit samples treated with propyl disulfide was conducted to observe the structural changes in these parts and to compare them with controls. The SEM micrographs of the various parts of the treated and control longan peel samples are presented in Figure 9. The exocarp is the outermost part of the longan peel. The propyl disulfide had a positive effect; as shown in Figure 9, the exocarps of the treated fruit samples were very smooth, complete, tight, and connected. This honeycomb-like structure was composed of cup-shaped cells that were uniform and more clear, while the exocarp of the control fruit was not complete, had free space, a loose structure, a non-uniform honeycomb appearance, less cup-shaped cells, and more mycelial pathogens. The mesocarp is the second layer of the longan peel and comprises about 70% of the longan pericarp. The mesocarp layer of the propyl-disulfide-treated fruit was more uniform and continuous than the control, where rough structures and many cracks and openings were observed. The rills were deeper and broader in control samples than treated samples. The endocarp is a single layer of cells. The endocarp layer of the propyl-disulfide-treated fruit showed a regular surface omamentation compared to the control fruit, where some irregular surface omamentation was observed.

## 4. Discussion

Water loss is one of the main reasons for pericarp browning in longan fruit, whereby shrinkage occurs when substrates, enzymes, and other cell constituents come into contact and initiate browning. This can be seen from the strong positive correlation of water loss with BI (r = 85) and L* values (r = 87), as shown in Table 3. Weight loss was significantly higher in control fruit samples than in all the other treatments. This is one of the benefits of polymeric films, which prevent water loss from fruit samples. On the other hand, PD also might play a role in water loss prevention, probably maintaining the pericarp’s integrity and reducing water loss. Additionally, it can be stated that PD worked as a barrier against water loss, as previously found in thymol-treated longan fruit samples [8] and fresh-cut beans treated with tea tree essential oil and peppermint essential oil [27].

PD effectively inhibited the growth of microbes on the longan fruit surfaces. Although DI percentages increased in all treatments at the end of storage, the DI percentage of the control longan fruit samples was comparatively higher than all other treatments (Figure 2). A possible mechanism of the decay prevention of PD could be attributed to the sulfur compound, as it is well known for its antimicrobial activity levels. Ramos et al. [28] reported that the antiadhesive mechanism of neem extract could be the hydrophobicity of the cell surfaces’ and the formation of biofilm, which could affect the microbial colonization. Koul [29] stated that either the characteristic odor of sulfur compounds or some physiological mechanism of interaction make the PD an effective grain protectant against insect pests. Kumar and Kudachikar [30] reported that the antifungal mechanisms of natural plant extracts against pathogens could be attributed to the disruption of membrane integrity and cellular component leakage. As microbial growth symptoms appear on the longan fruit’s surfaces, in the current research work we did not study the antimicrobial efficiency of PD on specific microorganisms, but generally evaluated the decay of longan fruit. Hence, no decay and less symptoms were found in PD-treated fruit samples than control fruit. The term decay incidence was used to describe this process, and the data are presented in Figure 3. However, in our previous study on mango fruit, we found that PD was very efficient in inhibiting the growth of major fungi in mango fruit samples. We found that PD effectively inhibited the mycelial growth of *Colletotrichum gloeosporioides* and *Colletotrichum acutatum*, causing anthracnose, as well as *Lasiodiplodia theobromae* and *Neofusicoccum parvum,* causing stem end rots in mango [14,15]. Future research should be directed toward the specific microorganisms found in longan fruit samples.

Pericarp browning of longan is another major concern that limits its postharvest life. The combined effect of PD and polymeric films delayed the pericarp browning. Pericarp browning is a complex phenomenon that may include many interconnected factors that can be represented in terms of color values, phenol contents, activity levels of enzymes, and so on. The effect of PD on browning inhibition was obvious in maintaining the color values of longan pericarp samples (Table 1). Compared to the control treatment, fruit samples treated with PD and packaged in various polymeric films had high L* values, lowest redness (a*), and high yellowness (b*). The presence of high TPC and TFC contents in longan pericarps treated with PD further confirmed the antioxidant efficiency of neem plant extracts. Interestingly, PD also affects the enzymes involved in the browning reaction. Longan fruit samples treated with PD and packaged in polymeric films showed lower PPO and POD activity levels than those in the control treatment. Regarding the overall phenomenon of the browning reaction and the antioxidant properties of neem, the high TPC and TFC contents (Figure 7), reduced activity levels of PPO and POD (Figure 8), high color values (Table 1), and lower BI levels (Figure 4) indicate the high antioxidant effect of PD. This can be seen from the correlation coefficient values in Table 3, whereby BI is highly correlated with PPO (r = 92), POD (r = 83), phenols (r = −85), flavonoids (r = −88), L* (r = −99), a* (r = 92), and b* (r = −93) (Table 3). This good fit of the correlation coefficients in these parameters and compounds involved in the enzymatic browning reaction and their dependency on each other shows the antioxidant efficiency of PD against the enzymatic browning in longan fruit samples. The apparent color changes in the longan pericarp further confirm the antioxidant activity of PD, as can be seen in Figure 6, where the longan fruit pericarp turned brown on day 9. Changes in PD-treated fruit and packaged in the polymeric films were very slow, and the fruit samples were still acceptable on day 18 in OPP-20 and OPP-40 packages. This trend of high phenols, reduced enzymes activity levels, and consequently low BI levels was also seen in our previous study, when longan fruit cv. Daw was treated with plant essential oil (thymol) [7,8]. Valero et al. [31] fumigated table grapes with thymol, eugenol, and menthol, and reported that these essential oils maintained the phenol contents and high color values better than the control. Similar to our study on the propyl disulfide compound, other phytochemicals, such as diallyl disulfide and diallyl trisulfide obtained from garlic essential oil, showed strong antioxidant activity against nicotinamide–adenine dinucleotide phosphate (NADPH) oxidase enzymes [32].

The antioxidant activity of neem extract was also studied regarding the preservation and shelf life extension of other food products. Ouerfelli et al. [19] preserved the quality and extended the shelf life of raw beef patties to 11 days storage at 4 °C. These authors reported that neem extract prevented the loss in color, reduced the metmyoglobin formation, scavenged the DPPH free radicals, and possessed high antibacterial potential against beef patties. Serrone and Nicoletti [18] preserved fresh retail meat using neem cake oil and reported its efficacy against a wide range of bacteria. Serrone et al. [11] reported that neem oil effectively preserved the quality of fresh retail meat.

The peel firmness of the longan was higher in the treated fruit than in control. As shown in Figure 6, the peel and aril breakdown in the control on day 9 meant they were not suitable for further storage and were discarded, while the complete aril and peel in the treated fruit showed that PD treatment and storage in various polymeric films, particularly OPP-20 and OPP-40, prevented softness and aril breakdown.

Longan fruit has a unique pericarp structure, and besides the effect of PD on the physicochemical characteristics, the impact of PD was also evaluated on the pericarp structure. The scanning electron microscopy analysis indicated that PD maintained a uniform smooth surface. The SEM analysis confirmed that the PD maintained the cell integrity and compactness, prevented water loss, and minimized the chance of pathogen growth. On the other hand, the pericarp of the control treatment was cracked and damaged, showing an irregular surface and free space (Figure 9). This kind of loose structure was beneficial for the pathogens’ invasion, as shown in Figure 3 (decay incidence), and enhanced the water loss, as shown in Figure 2.

Another reason for the good quality of the longan fruit being maintained could be the application of PD in the vapor phase due to the slow release time, which might maintain the quality and suppress the microbes for a longer time. Another benefit of the vapor phase application is that low quantities of PD are required, without altering the sensory properties of the products. Hence, the shelf life of the longan fruit treated with PD was extended up to 18 days compared to 9 days in control.

## 5. Conclusions

In this study, we applied a new fumigation method by placing the essential oil on the sterile gauze inside the polymeric films. Fruit samples without PD and in open net packages were considered as controls and all packages were stored at 12 ± 1 °C for 18 days. In combination with plant extract (PD), polymeric films played a very effective role in the quality maintenance of longan fruit samples. Compared to control packages, all types of polymeric films in combination with PD prevented weight loss, inhibited microbial growth, and delayed the pericarp browning of longan fruit samples. PD-treated fruit samples and sealed in packaging films also exhibited high firmness and color values, better prevented the oxidation of the phenols and flavonoids, and better inhibited enzyme activity than the control treatment. Extended shelf life of 18 days was observed in OPP-20 and OPP-40 packaging films with good quality attributes. SEM analysis showed a clear uniformity in the pericarp structures, which was entirely associated with the obtained results. Overall, PD maintained the structural integrity of the pericarp, prevented pericarp browning, inhibited the growth of microorganisms, and had a very positive effect on longan fruit quality as compared to control fruit samples. Therefore, with a combination of OPP-20 and OPP-40 polymeric films, PD could be applied commercially as a potent antioxidant and antimicrobial agent in a wide range of food products to replace synthetic fumigants or preservatives.

## Figures and Tables

**Table 1:** Color values of longan pericarps fumigated with propyl disulfide in different packaging films in comparison to control, stored fruit at 12 ± 1 °C.

Treatments							
Days	OPP-20	OPP-40	OPP-60	PE-20	PE-40	PE-60	Control
L*							
0	69.44 ^aAB^ (2.44)	69.44 ^aA^ (2.44)	69.44 ^aA^ (2.44)	69.44 ^aA^ (2.44)	69.44 ^aA^ (2.44)	69.44 ^aA^ (2.44)	69.44 ^aA^ (2.44)
3	70.68 ^aA^ (0.26)	67.47 ^abAB^ (1.90)	66.25 ^bAB^ (1.70)	65.64 ^bB^ (0.89)	67.13 ^abA^ (2.30)	64.74 ^bA^ (0.78)	56.59 ^cB^ (2.22)
6	66.81 ^aBC^ (0.14)	67.00 ^aAB^ (0.17)	66.91 ^aAB^ (0.06)	66.22 ^aB^ (0.87)	66.61 ^aA^ (0.42)	65.84 ^aA^ (1.10)	48.44 ^bC^ (2.44)
9	66.01 ^aCD^ (0.51)	66.47 ^aB^ (0.61)	64.33 ^bB^ (2.23)	61.88 ^cC^ (2.07)	62.16 ^cB^ (1.14)	59.39 ^dB^ (0.86)	35.50 ^eD^ (0.35)
12	62.70 ^aDE^ (0.28)	64.89 ^aB^ (0.82)	56.91 ^bC^ (0.06)	53.55 ^bD^ (0.13)	53.27 ^bC^ (2.61)	52.28 ^bC^ (4.16)	–
15	59.37 ^aEF^ (1.15)	60.61 ^aC^ (0.73)	48.02 ^bD^ (1.13)	46.88 ^bE^ (0.93)	48.83 ^bD^ (2.82)	–	–
18	57.94 ^aE^ (0.16)	56.03 ^aD^ (1.02)	–	–	–	–	–
	a*						
0	1.78 ^aC^ (0.24)	1.78 ^aE^ (0.24)	1.78 ^aD^ (0.24)	1.78 ^aE^ (0.24)	1.78 ^aD^ (0.24)	1.78 ^aD^ (0.24)	1.78 ^aD^ (0.24)
3	2.36 ^bC^ (0.22)	3.44 ^aD^ (0.30)	2.64 ^bC^ (0.21)	4.00 ^aD^ (0.15)	3.38 ^aC^ (0.29)	3.61 ^aBC^ (0.19)	3.45 ^aC^ (0.13)
6	4.02 ^abcB^ (0.08)	3.65 ^bcCD^ (0.28)	3.84 ^abcB^ (0.23)	4.23 ^abCD^ (0.33)	3.83 ^abcC^ (0.21)	3.38 ^cC^ (0.26)	4.51 ^aB^ (0.26)
9	4.11 ^bcB^ (0.28)	4.23 ^bcBCD^ (0.06)	4.12 ^bcB^ (0.13)	4.68 ^bBC^ (0.20)	4.06 ^cBC^ (0.24)	4.23 ^bcB^ (0.16)	5.87 ^aA^ (0.35)
12	4.43 ^bB^ (0.10)	4.51 ^bBC^ (0.10)	4.57 ^abB^ (0.16)	4.91 ^abAB^ (0.18)	4.57 ^abB^ (0.18)	5.23 ^aA^ (0.17)	–
15	4.68 ^bAB^ (0.03)	5.07 ^abAB^ (0.24)	6.02 ^aA^ (0.19)	5.58 ^abA^ (0.43)	5.35 ^abA^ (0.17)	–	–
18	5.19 ^bA^ (0.19)	5.84 ^aA^ (0.33)	–	–	–	–	–
	b*						
0	35.23 ^aA^ (0.85)	35.23 ^aA^ (0.85)	35.23 ^aA^ (0.85)	35.23 ^aA^ (0.85)	35.23 ^aA^ (0.85)	35.23 ^aA^ (0.85)	35.23 ^aA^ (0.85)
3	33.96 ^bA^ (0.10)	35.04 ^aA^ (0.35)	30.29 ^bB^ (1.82)	28.66 ^bcB^ (0.75)	35.15 ^aA^ (0.34)	26.14 ^cdB^ (1.90)	23.82 ^dB^ (2.14)
6	31.51 ^aAB^ (0.62)	32.15 ^aB^ (0.34)	27.74 ^bBC^ (0.59)	26.66 ^bcBC^ (1.14)	32.37 ^aA^ (0.25)	23.92 ^cdBC^ (0.67)	20.38 ^dC^ (1.32)
9	28.51 ^aBC^ (0.93)	30.60 ^aB^ (0.91)	25.40 ^bC^ (0.80)	25.33 ^bC^ (0.22)	28.71 ^aB^ (0.60)	20.80 ^cCD^ (1.29)	18.05 ^dC^ (0.57)
12	25.74 ^aCD^ (0.70)	25.71 ^aC^ (0.96)	20.96 ^bD^ (1.33)	22.55 ^abD^ (1.08)	26.15 ^aB^ (0.09)	18.92 ^bD^ (0.51)	–
15	22.96 ^aDE^ (1.59)	22.37 ^aD^ (0.23)	18.51 ^bD^ (0.26)	21.33 ^aD^ (0.59)	21.37 ^aC^ (1.05)	–	–
18	20.96 ^aE^ (0.37)	19.71 ^bE^ (0.21)	–	–	–	–	–

Different small letters in each row and capital letters in each column show the significant differences between the treatments within the same day and between the days in the same treatment, respectively, via LSD test at *p* ≤ 0.05. SDs are presented in parenthesis.

**Table 2:** TSS, TA, and pH values of longan fruit treated with propyl disulfide in different packaging films in comparison to control, stored samples at 12 ± 1 °C.

Treatments							
Days	OPP-20	OPP-40	OPP-60	PE-20	PE-40	PE-60	Control
	TSS						
0	20.73 ^aA^ (0.12)	20.73 ^aA^ (0.12)	20.73 ^aA^ (0.12)	20.73 ^aA^ (0.12)	20.73 ^aA^ (0.12)	20.73 ^aA^ (0.12)	20.73 ^aA^ (0.12)
3	18.77 ^bcC^ (0.15)	18.53 ^cC^ (0.32)	18.40 ^cC^ (0.10)	18.80 ^bcB^ (0.40)	18.60 ^cD^ (0.26)	19.17 ^abC^ (0.29)	19.43 ^aB^ (0.38)
6	19.10 ^bB^ (0.10)	18.10 ^cC^ (0.10)	17.13 ^eE^ (0.15)	17.73 ^dC^ (0.15)	20.07 ^aB^ (0.06)	18.23 ^cD^ (0.12)	19.13 ^bBC^ (0.12)
9	18.90 ^cBC^ (0.20)	19.13 ^bcB^ (0.15)	19.50 ^abB^ (0.17)	17.93 ^dC^ (0.21)	19.03 ^cC^ (0.06)	19.90 ^aB^ (0.35)	18.87 ^cC^ (0.35)
12	18.97 ^aBC^ (0.23)	17.13 ^cdD^ (0.47)	13.63 ^bcD^ (0.31)	16.97 ^dD^ (0.15)	17.93 ^bE^ (0.35)	17.90 ^bD^ (0.17)	–
15	18.70 ^aC^ (0.30)	17.50 ^bD^ (0.36)	17.47 ^bD^ (0.15)	16.83 ^cD^ (0.25)	17.47 ^bF^ (0.21)	–	–
18	17.53 ^aD^ (0.15)	17.27 ^aD^ (0.55)	–	–	–	–	–
	TA						
0	0.15 ^aD^ (0.01)	0.15 ^aE^ (0.01)	0.15 ^aE^ (0.01)	0.15 ^aE^ (0.01)	0.15 ^aC^ (0.01)	0.15 ^aD^ (0.01)	0.15 ^aB^ (0.01)
3	0.16 ^bcC^ (0.01)	0.16 ^bcD^ (0.01)	0.17 ^abD^ (0.01)	0.16 ^bcD^ (0.01)	0.15 ^cC^ (0.01)	0.17 ^abC^ (0.01)	0.18 ^aA^ (0.01)
6	0.19 ^aB^ (0.01)	0.17 ^bD^ (0.01)	0.19 ^aC^ (0.01)	0.16 ^bD^ (0.01)	0.18 ^aB^ (0.01)	0.19 ^aB^ (0.01)	0.18 ^aA^ (0.01)
9	0.19 ^bB^ (0.01)	0.19 ^bC^ (0.01)	0.21 ^aB^ (0.01)	0.18 ^bC^ (0.01)	0.20 ^abB^ (0.01)	0.20 ^abB^ (0.01)	0.17 ^cA^ (0.01)
12	0.20 ^bB^ (0.01)	0.20 ^bBC^ (0.01)	0.22 ^aAB^ (0.01)	0.20 ^bB^ (0.01)	0.22 ^aA^ (0.01)	0.23 ^aA^ (0.01)	–
15	0.23 ^abA^ (0.01)	0.21 ^cAB^ (0.01)	0.23 ^abA^ (0.01)	0.22 ^bcA^ (0.01)	0.24 ^aA^ (0.01)	–	–
18	0.23 ^aA^ (0.01)	0.22 ^aA^ (0.01)	–	–	–	–	–
	pH						
0	6.88 ^aG^ (0.02)	6.88 ^aE^ (0.02)	6.88 ^aE^ (0.02)	6.88 ^aE^ (0.02)	6.88 ^aE^ (0.02)	6.88 ^aD^ (0.02)	6.88 ^aD^ (0.02)
3	6.94 ^abcF^ (0.02)	6.96 ^abcD^ (0.05)	6.93 ^bcD^ (0.03)	6.92 ^cE^ (0.05)	6.98 ^aD^ (0.02)	6.97 ^abC^ (0.02)	6.99 ^aC^ (0.01)
6	6.99 ^cE^ (0.01)	6.99 ^cD^ (0.01)	6.97 ^cC^ (0.02)	6.98 ^cD^ (0.01)	7.11 ^bC^ (0.02)	7.11 ^bB^ (0.02)	7.20 ^aB^ (0.02)
9	7.13 ^cdD^ (0.04)	7.18 ^bC^ (0.02)	7.10 ^dB^ (0.01)	7.14 ^cC^ (0.02)	7.25 ^aB^ (0.02)	7.10 ^cdB^ (0.01)	7.29 ^aA^ (0.03)
12	7.20 ^cC^ (0.01)	7.26 ^bB^ (0.03)	7.33 ^aA^ (0.03)	7.21 ^cB^ (0.02)	7.25 ^bcB^ (0.05)	7.27 ^bA^ (0.02)	–
15	7.31 ^bB^ (0.02)	7.31 ^abA^ (0.03)	7.32 ^abA^ (0.02)	7.26 ^cA^ (0.03)	7.35 ^aA^ (0.01)	–	–
18	7.36 ^aA^ (0.03)	7.31 ^bAB^ (0.02)	–	–	–	–	–

Different small letters in each row and capital letters in each column show the significant differences between the treatments within the same day and between the days in the same treatment, respectively, via LSD test at *p* ≤ 0.05. SDs are presented in parentheses.

**Table 3:** Pearson’s correlation coefficient values for different quality parameters in longan fruit samples stored at 12 ± 1 °C.

	Wt. Loss	BI	DI	Texture	L*	a*	b*	TSS	TA	pH	TPC	TFC	PPO	POD
Wt. loss	1													
BI	0.87	1												
DI	0.68	0.94	1											
Texture	−0.47	−0.79	−0.90	1										
L*	−0.85	−0.99	−0.96	0.79	1									
a*	0.70	0.92	0.95	−0.90	−0.92	1								
b*	−0.71	−0.93	−0.94	0.87	0.92	−0.99	1							
TSS	−0.48	−0.80	−0.91	0.93	0.80	−0.96	0.94	1						
TA	0.31	0.70	0.83	−0.91	−0.69	0.89	−0.89	−0.97	1					
pH	0.63	0.89	0.92	−0.91	−0.87	0.98	−0.98	−0.96	0.93	1				
TPC	−0.59	−0.85	−0.89	0.93	0.83	−0.97	0.97	0.95	−0.94	−0.99	1			
TFC	−0.61	−0.88	−0.94	0.93	0.88	−0.99	0.98	0.97	−0.94	−0.99	0.99	1		
PPO	0.64	0.92	0.99	−0.91	−0.93	0.95	−0.94	−0.92	0.86	0.92	−0.90	−0.95	1	
POD	0.51	0.83	0.96	−0.93	−0.86	0.93	−0.91	−0.96	0.91	0.91	−0.89	−0.94	0.97	1

## Data Availability

Not applicable.
